#  Situações difíceis e sentimentos no cuidado paliativo oncológico 

**DOI:** 10.1590/0102-311XPT116823

**Published:** 2024-02-09

**Authors:** Vanessa dos Santos Beserra, Claudia Brito

**Affiliations:** 1 Instituto Nacional de Câncer José de Alencar Gomes da Silva, Rio de Janeiro, Brasil.; 2 Escola Nacional de Saúde Pública Sergio Arouca, Fundação Oswaldo Cruz, Rio de Janeiro, Brasil.

**Keywords:** Cuidados Paliativos, Neoplasias, Sentimentos, Profissionais de Saúde, Palliative Care, Neoplasms, Feelings, Health Care Professionals, Cuidados Paliativos, Neoplasias, Sentimientos, Profesionales de la Salud

## Abstract

O ato de cuidar cotidianamente de pessoas com dor, falta de ar e em morte iminente pode potencializar situações difíceis para profissionais da área. Contudo, raramente são discutidas nos serviços e no processo de formação profissional. Objetivou-se, então, analisar situações difíceis e sentimentos que emergem do cuidado de saúde. Esta é uma pesquisa de perspectiva fenomenológica e qualitativa, baseada em 30 situações difíceis de profissionais de saúde que atuam exclusivamente no cuidado paliativo oncológico. As entrevistas foram realizadas de agosto de 2019 a fevereiro de 2020. Os resultados mostram que as principais dificuldades foram motivadas pela identificação (quando o profissional vê semelhança com o paciente que cuida), morte ruim (com sofrimento), quando o paciente era jovem, morte de mãe com filho pequeno e quando havia divergência entre o proposto pelo profissional e a recusa do paciente. Percebeu-se relação entre tipos de situações difíceis e categoria profissional. Os profissionais expressaram tanto sentimentos desagradáveis (tristeza, impotência, angústia, medo) quanto agradáveis (compaixão, gratidão). Os resultados mostram que o ocultamento do processo de morrer ao longo do desenvolvimento civilizatório transformou-o em tabu, angustiante inclusive para quem trabalha com cuidados paliativos. Contribuem, também, para mostrar uma importante dimensão subjetiva do cuidado, geralmente negligenciada, que gera sofrimento, mas também ressignificação. Para que alguém cumpra seu propósito, é necessário encontrar sentido no trabalho, possibilitado pela modificação do estado interno do profissional pela experiência, que gera transformação e novo significado e saber a partir da práxis.

## Abstract (en)

The act of caring for people in pain, shortness of breath, and imminent death on a daily basis can intensify difficult situations for health professionals. However, difficult situations are rarely discussed in the services and in the professional training process. This study aimed to analyze difficult situations and feelings that emerge from healthcare. This is a phenomenological and qualitative study based on 30 difficult situations of health professionals who work exclusively in palliative oncological care. The interviews were conducted from August to February 2020. The results show that the main difficulties were motivated by identification (when the professional recognizes similarities in the patient they are caring for), a bad death (with suffering), caring for young patients, the death of a mother with a small child, and when there was a divergence between what the professional proposed and the patient’s choice. There was a relationship between types of difficult situations and professional category. The professionals expressed both unpleasant feelings (sadness, impotence, anguish, fear) and pleasant feelings (compassion, gratitude). The results show that the concealment of the dying process throughout the societies’ development turned it into a taboo, causing distress even in those who work in palliative care. They also show an important subjective dimension of care, usually neglected, which generates suffering, but also resignification. To properly provide care, health professionals must find meaning in the work, made possible by the modification of the their internal mindset via experience, which generates transformation, new meaning, and knowledge from praxis.

## Abstract (es)

El acto de atender diariamente a personas con dolor, dificultad para respirar y muerte inminente puede potenciar situaciones difíciles para los profesionales del área. Sin embargo, casi nunca se discuten en los servicios y en el proceso de formación profesional. El objetivo fue analizar situaciones difíciles y sentimientos que emergen del cuidado de la salud. Investigación de perspectiva fenomenológica y cualitativa, basada en 30 situaciones difíciles de profesionales de la salud que actúan exclusivamente en el cuidado paliativo oncológico. Las entrevistas se realizaron entre agosto y febrero de 2020. Los resultados enseñan que las principales dificultades se motivaron por la identificación (cuando el profesional ve similitud con el paciente que atiende), mala muerte (con sufrimiento), cuando el paciente era joven, muerte de una madre con hijo pequeño y cuando hubo discrepancia entre lo propuesto por el profesional y el rechazo del paciente. Se observó una relación entre tipos de situaciones difíciles y categoría profesional. Los profesionales expresaron tanto sentimientos desagradables (tristeza, impotencia, angustia, miedo) como agradables (compasión, gratitud). Los resultados indican que ocultar el proceso de morir a lo largo del desarrollo de la civilización lo convirtió en un tabú e, incluso, es angustiante para las personas que trabajan con cuidados paliativos. También contribuye a mostrar una importante dimensión subjetiva del cuidado, generalmente ignorada, que genera sufrimiento, pero también resignificación. Para que cumpla con su propósito es necesario encontrar sentido en el trabajo, posibilitado por la modificación del estado interno del profesional a través de la experiencia, generando transformación y un nuevo significado y conocimiento a partir de la praxis.

## Introdução

O cuidado paliativo é uma abordagem destinada às pessoas com doenças fatais, de qualquer idade, sem perspectiva curativa, buscando qualidade no tempo restante de vida ^1^. Estima-se que, a cada ano, mais de 56,8 milhões de pessoas necessitarão desse tipo de cuidado ^1^. O trabalho do profissional nessa área visa aliviar a dor e o sofrimento ^2^, ou proporcionar uma “boa morte”, no jargão desses trabalhadores.

Cerca de 80% dos pacientes oncológicos, em cuidados ao fim de vida, sentem dor intensa e dispneia, necessitando de opioides para seu controle ^2^. É recomendado que os profissionais, na administração da morfina, encontrem equilíbrio entre lucidez e sedação paliativa para o paciente, visando à autonomia e capacidade de interação do sujeito ^3^. Ainda com relação à dor, desde as décadas de 1950-1960, Cicely Saunders, precursora da área, já alertava que não era apenas uma manifestação física, pois havia também uma dimensão psicossocial e espiritual ^4^. Siebers ^5^ contribuiu mostrando a dimensão sociocultural, tendo em vista que a dor do outro gera sofrimento naquele que o observa, pois representa uma fonte de terror no imaginário social, por afetar a dignidade humana. E complementa dizendo que a dor pode ser um gatilho para fortes emoções, opiniões e julgamentos ^5^.

Outro desafio do cuidado paliativo é o processo de morrer e a morte em si. Sobre isso, Elias ^6^ mostrou inúmeras mudanças ocorridas durante o desenvolvimento civilizatório que propiciaram o afastamento da morte - e dos moribundos - no cotidiano dos sujeitos e, por conseguinte, a dificuldade de lidar com ela de forma natural. Um dos exemplos foi o incentivo de suprimir os sentimentos, sobretudo aqueles fortes e espontâneos, diante dos moribundos. Isso provocou, na sociedade, o dilema de não saber lidar com a morte, da dificuldade para encontrar a palavra certa ou o gestual adequado e do receio de criar situações embaraçosas ^6^.

O ato de cuidar cotidianamente de pessoas com dor, falta de ar e em morte iminente, pode potencializar situações difíceis para os profissionais da área. Apesar disso, essas questões raramente são colocadas em análise ou discutidas no exercício clínico e no processo de formação profissional. Na prática, tais situações produzem desconforto, embaraço, falta de espontaneidade e afastamento ^6^, constituindo o tabu de que para prestar um cuidado apropriado, os sentimentos não podem ser explicitados.

O diagnóstico de câncer, além de ser muito frequente, estimado em 483 mil novos casos/ano no triênio 2023-2025 (excluindo câncer de pele não melanoma), no Brasil ^7^, é igualmente estigmatizado por sua associação com a morte inevitável, da qual, inclusive, evita-se falar o nome ^8^. Essa falta de diálogo associada a mecanismos de automatização das tarefas profissionais, não incomuns no cotidiano, pode redundar em sofrimento físico e psíquico do paciente e do profissional.

Nesse sentido, não apenas a ocorrência da morte em si, mas todo o percurso do cuidado paliativo oncológico pode envolver sentimentos exacerbados e recalcados pelos profissionais. Estamos denominando essas experiências como “situação difícil”.

Essa pesquisa objetivou analisar as situações difíceis e os sentimentos que emergem do cuidado, na perspectiva do profissional de saúde da prática paliativa oncológica.

## Método

Pesquisa de perspectiva compreensiva e fenomenológica, baseada na experiência ^9^ de situações difíceis por profissionais de saúde que prestavam cuidado paliativo a pacientes oncológicos internados ou a seus respectivos acompanhantes. Este estudo ocorreu na área de internação de uma unidade pública hospitalar, exclusiva de cuidados paliativos oncológicos.

Tendo em vista que esta pesquisa é oriunda de experiência profissional, utilizou-se o conceito de Larrosa, que enfatiza que a experiência não é informação, não é opinião, e sim “*o que nos passa, o que nos acontece, o que nos toca*” ^9^ (p. 18). Para Larrosa ^9^, a mudança do estado interno do sujeito no cotidiano do cuidado possibilita a experiência, que gera uma transformação e um novo significado e saber a partir da práxis.

Partiu-se do pressuposto de que uma pluralidade de condições (internas e externas) têm o potencial de gerar uma situação difícil, assim, não houve exclusão por categoria profissional ou tempo de serviço, apenas aqueles em férias ou licença durante o período do estudo ou que estavam em funções administrativas não foram entrevistados. Como resultado, foram incluídos profissionais de saúde que atuavam diretamente no cuidado paliativo a pacientes oncológicos internados.

O convite para a entrevista iniciou-se por meio de aplicativo de conversa individualizado, para alguns membros da equipe multiprofissional. Os convidados também sugeriram outros profissionais para serem entrevistados. Adicionalmente, alguns, ao tomarem ciência da pesquisa, demonstraram interesse em participar e foram incluídos. Destaca-se que o grande interesse dos trabalhadores em compartilhar suas experiências foi inesperado e denota o quanto o tema é relevante e pouco abordado no serviço de saúde. Nesse sentido, a seleção foi orientada pela motivação do trabalhador, como força motriz dessa inclusão.

As entrevistas foram realizadas na unidade, no período de agosto de 2019 a fevereiro de 2020, em local de acesso restrito. As experiências narradas foram gravadas, transcritas e revisadas. Partiu-se de 36 entrevistados; entretanto, após análise inicial, seis entrevistas foram excluídas. Os motivos de exclusão foram: abordarem o tema de forma teórica ou hipotética; narrarem exclusivamente problemas de gestão ou relacionamento de equipe, não evidenciando as situações difíceis na prestação do cuidado. Assim, permaneceram 30 entrevistas (situações difíceis), das seguintes categorias profissionais: técnicos de enfermagem, enfermeiros, médicos, fisioterapeutas e assistentes sociais. O quantitativo de entrevistados representava 22% do total de profissionais da unidade de internação.

As entrevistas priorizaram as seguintes questões norteadoras: (a) O que aconteceu no cuidado que foi difícil para você e por que ela foi difícil?; e (b) Quais sentimentos e emoções você teve a partir dessa situação?

O método investigativo fenomenológico busca a compreensão do que acontece com o sujeito na sua interação com o mundo e o sentido que ele dá às situações que vive, ou seja, na pesquisa em questão, as situações difíceis do cuidado paliativo.

Assim, a análise dos dados foi orientada por três momentos: descrição, redução e compreensão. De forma resumida, procedeu-se à seleção dos aspectos essenciais das descrições, pondo em destaque todas as afirmações relativas às vivências, para sistematizar (redução) os resultados por meio do agrupamento dos núcleos de sentidos e, então, compor as categorias (situações difíceis e sentimentos) e analisá-las para a compreensão do fenômeno. Nesse sentido, a compreensão fenomenológica é possibilitada pela transposição dos achados individuais para o geral, que resulta das convergências, divergências e peculiaridades que se apresentam nos casos individuais.

Todas as entrevistas compartilhadas apresentaram mais de um tipo de situação difícil e sentimento. Os Quadros 1 e 2 apresentam os agrupamentos realizados, a definição das respectivas categorias no contexto desta pesquisa e verbetes da narrativa dos profissionais de saúde entrevistados.

**Quadro 1:** Tipos, definição e relatos de situações difíceis.

CATEGORIA	DEFINIÇÃO	RELATOS DOS ENTREVISTADOS
Identificação	Profissional associa algo do cuidado com sua vida particular	“*As* [situações] *que mais tenho dificuldade é exatamente prestar cuidado a pacientes mais idosos, porque na verdade eu me coloco no lugar, porque a imagem do idoso remete à minha mãe.* (...) *essas são as de maior potência para me mobilizar*” (Entrevista 2, Enfermeira) “*Ela tinha um biotipo, a estatura parecida, cabelo preto.* (...) *passou pela minha cabeça exatamente de que poderia ser eu. Assim, qual é a diferença? Nenhuma. Inclusive, você começa a pensar o que faria se você estivesse no lugar dela*” (Entrevista 12, Médica)
Paciente jovem	Remete a uma vida que não pode ser vivida, sonhos não concretizados e suscita questionamentos existenciais sobre “justiça divina”, como se não merecesse tal sofrimento pelo tempo de vida	“*Ele tem 25 anos,* (...) *tem um mundo pela frente e deixar uma filha de 2 anos, uma esposa maravilhosa* (...) *me pergunto: até que ponto o ser humano merece estar passando por aquilo?*” (Entrevista 1, Enfermeira) “*Um rapaz de 19 anos que tinha acabado de passar em um concurso que era uma coisa que ele queria MUITO, e descobriu um câncer* (...)*,* [a mãe] *planejou tantas coisas com ele, festejaram a vitória* [concurso] *e não iam poder curtir aquele momento*” (Entrevista 16, Enfermeira)
Tumor de crescimento rápido	Tumores de evolução rápida, sem dar chances de um tratamento da doença	“*Em situações tipo sarcoma, que você não tem a menor possibilidade, diferente do colo de útero, de mama, que você ainda pode pegar no início e que você pode fazer alguma coisa* (...)*. Ali era assim: quer queira, quer não, seu destino está traçado!*” (Entrevista 12, Médica)
Morte ruim	Morte com intensa agonia (asfixia; com contenção mecânica; extravasamento de fluidos), vivenciada pelos profissionais como impossibilidade de prover uma boa morte	“*Ela começou a gritar dizendo que estava vendo as pessoas indo buscá-la: ‘Eles vieram me buscar! Eles vieram me buscar!’ Ela conseguiu me puxar, eu CAÍ em cima dela na cama, eu fiquei caída, tocando aquela campainha, gritando... eu tentando empurrar ela, e ela me agarrando,* (...) *nisso a gente fez os* [sedativos]*. Ela CONTINUOU aos gritos, ela morreu desse jeito!*” (Entrevista 7, Enfermeira) “*Ele começou a sentir MUITA, MUITA falta de ar e* (...) *era uma cena de terror, ele não conseguia nem falar direito, agoniado* (...) *e falava: ‘ai meu Deus, não me leva, eu não quero morrer’ e a mãe chorando, segurando a mão dele.* (...) *e ele olhando pra mim, falava: ‘eu tô indo embora, eu vou morrer’* (...) *ele podia só morrer, não precisava morrer desse jeito*” (Entrevista 23, Enfermeira)
Medicamento sem efeito	Retardo do efeito da medicação em situação de agonia dos pacientes	“*Dei a dose extra da morfina* (...) *e nada dele melhorar, pelo contrário, só piorava* (...) *fiz o sedativo SOS de novo, parecia água com açúcar... não respondeu nada, totalmente acordado* (...) *quando ele dormiu, abracei a mãe e ela falou: ‘eu quero que Deus leve logo ele pra acabar com isso’*” (Entrevista 23, Enfermeira)
Binômio mãe-filho	Quando mãe presencia morte de filho(a) ou filho(a) pequeno(a) presencia morte de mãe	“*A filha, de uns 7 anos, veio visitá-la. Me lembro que ela não queria entrar no quarto para ver a mãe* (...) *eu falei assim: ‘Ah...* [choro] [silêncio prolongado] *você não quer entrar?’* [voz embargada] *e a menina: ‘Não tia, eu não quero ver minha mãe assim’ e falei: ‘Mas, você não quer ir lá, dar um beijo na sua mãe?’ e ela perguntou: ‘Tia, minha mãe vai morrer?’, daí eu respondi: ‘Então... morrer né?... eu sei que é difícil. Você é muito pequena, mas um dia todo mundo vai morrer, é assim, é o ciclo da vida.’ E ela: ‘É tia, mas podia morrer todo mundo no mesmo dia, né? Porque a gente não ia ficar com saudade’, aí falei* [voz embargada]*: ‘Nossa! você tem razão, eu nunca tinha parado pra pensar nisso!’ E ela chorando: ‘Eu posso dar um beijo na minha mãe?’ E eu: ‘Pode!’ E ela: ‘Você diz para ela que eu amo muito ela?’. Então eu: ‘Mas diz você!’. E entrei com ela, abaixei a cama e ela chorando muito, deu um beijo na mãe. Aquilo ali para mim... ver aquele sentimento, aquela menininha, me marcou para o resto da vida*” (Entrevista 15, Enfermeira) “*A paciente estava numa crise de ansiedade absurda, aí perguntei: ‘Por que você tá tão ansiosa?’ e ela: ‘eu tenho que ir embora, eu tenho que ir embora! Eu tenho meu filho pequeno,* [que] *me ligou ontem, falou pra mim: mamãe, eu rezo todo dia pra papai do céu pra você voltar pra casa e não ir morar no céu. Mamãe, se você morar no céu, eu vou ter que ir morar no céu também’.* (...) *hoje, se você me perguntar o que mais me afeta:* (...) *mães jovens que estão vivendo a sua finitude e vão deixar filhos pequenos*” (Entrevista 18, Fisioterapeuta)
Perspectiva curativa trabalhando em cuidados paliativos	Profissional que atua na unidade exclusiva de cuidados paliativos, porém, ainda com uma intenção curativa	“*Eu não podia fazer mais nada pra ajudar, pra que ela ficasse bem, pra que ela saísse daquela situação.* (...) *e, naquele dia, último dia de vida dela, eu fiz tudo que eu podia, mas não era mais suficiente.* (...) *eu fiquei muito triste. Eu fui ali pra escada chorei até morrer* [risos]*, chorei muito*” (Entrevista 5, Técnica em Enfermagem) “*Quando eu estou em um hospital que não é paliativo, eu sei que vou intubar, botar na ventilação mecânica* (...)*, é a sensação de que eu realmente estou fazendo de tudo.* (...) *no paliativo, a gente não vai fazer isso, e chega um momento, que realmente, não tem mais o que fazer, assim, sempre tem algo a fazer, mas não ativamente*” (Entrevista 22, Médica)
Divergência de conduta	Quando o profissional não concorda com a conduta de outro profissional ou com a decisão de paciente/família	“*Foi um paciente* (...) *não estava mais controlando a dispneia, e quando chegou o PIOR dia, era um PEIXE fora d’água, ele recusou o dripping* [infusão contínua]*.* (...) *Quando eu cheguei no quarto com a bomba* [infusora]*, ele já sabia, pois, a médica já tinha falado que ia começar o dripping. Ele olhou para mim, o olhar dele foi de medo e falou: ‘Não, eu não quero! eu não quero isso!’. Eu vi nos olhos dele que ele enxergou a morte naquela máquina e, sofrendo, sofrendo, sofrendo, ele não quis. Eu tentei argumentar, tentei falar e ele, lúcido e orientado: ‘NÃO, não e não!’.* (...) *Eu sei que foi uma decisão dele, mas eu não consegui fazer mais por ele, queria ter feito mais*” (Entrevista 11, Enfermeira) “*Um caso que me marcou muito foi de um idoso, lúcido, orientado, acamado e muito consumido pela doença. Durante o atendimento, manifestou desejo de se CASAR com a companheira com quem ele tinha três dos seus quatro filhos, porém, dos três filhos biológicos do casal, dois não concordavam com o casamento, pois tinham algum conflito com a mãe.* (...) *Isso gerou uma discussão muito importante, porque a equipe deu uma ‘rachada’ no sentido de: ‘Pera aí, mas a gente tinha que ter a autorização dos filhos’ e ‘Não, mas o paciente é lúcido e orientado!’. Então, como organizar o cuidado para um paciente lúcido e orientado? Porque, às vezes, a gente acha que por estar acamado, ele perdeu sua autonomia de escolha*” (Entrevista 14, Assistente social)
Desfiguração da face	Deformidades de grande dimensão na face causadas pelo tumor	“*Foi um paciente com miíase* [larvas] *em lesão tumoral extensa de face, muito tecido necrosado* (...) *tudo era tumor* (...) *as únicas áreas que permaneciam intactas eram boca e nariz.* (...) *Quando eu tentava fazer o curativo, partes do tecido do rosto dele iam saindo nas minhas mãos*” (Entrevista 24, Enfermeira) “*Foi uma paciente com carcinoma de cavidade oral* (...) *gigante, ulcerada, que sangrava muito.* (...) *o esposo me mostrou as fotos dela, de poucos meses atrás, e era uma incrível DESFIGURAÇÃO, isso me comoveu muito* (...)*, principalmente pela velocidade...*” (Entrevista 22, Médica)
Paciente bonzinho	Paciente que não reclama de nada e somente agradece, inclusive em situações de intenso desconforto ou sofrimento	“*Ele era muito tranquilo* (...)*, muito bonzinho e isso até pesa mais, você pensa: ‘o garoto não reclama de nada, pra ele tá tudo bom, agradece o tempo todo’* (...) *e você se pergunta: ‘por que está sofrendo isso?’*” (Entrevista 23, Enfermeira) “*Foi um paciente* (...) *muito, muito simpático, atencioso,* (...) *e eu me apeguei a ele, envolvia todo mundo, não tinha como não se envolver.* (...) *mas ele foi evoluindo,* [e com a] *piora do quadro, acabou vindo a óbito comigo.* (...) *nesse dia ele ficou pedindo muito resgate* [dose extra] *de morfina e de* [sedativo]*, porque ele tava muito dispneico, cansado. E toda vez que ele pedia, ele pedia desculpa porque tava incomodando. E aí aquilo me incomodou muito, entendeu?*” (Entrevista 30, Técnica em Enfermagem)
Violência contra a equipe	Insultos verbais ou agressões físicas sofridas pelos profissionais de saúde	“*A gente estava* [fazendo procedimento] *na paciente super grave e a filha olhando pra mim, ela dizia: ‘Eu conheço gente do seu tipo, você não vale nada’. E, assim, eu realmente não entendi, porque eu não fiz expressão nenhuma, a gente não falou nada, porque a gente estava mesmo calada. E ela começou a gritar, assim, ela descompensou total e eu sem saber o que fazer*” (Entrevista 3, Técnica em Enfermagem) “*Eu fui fazer os cuidados do paciente* (...) *e, a filha dele* (...)*: ‘O que você está fazendo no meu pai? Que medicação é essa?’. Só que a forma que ela me abordou, eu não gostei, achei muito grosseira.* (...) *Eu já tinha terminado, e falei: ‘No momento eu não estou fazendo nenhuma medicação, quando eu terminar* [o procedimento]*, eu vou te falar* (...) *e* [a acompanhante]*: ‘Você é muito mal-educada porque eu te perguntei e você não me respondeu’.* (...) *eu virei pra ela e falei* (...)*: ‘Eu não vou fazer a medicação do seu pai, vou pedir pra vir outra pessoa fazer’* (...) *Ela continuou QUESTIONANDO por que eu não quis responder e sai* (...)*, nisso, ela voltou tirando minha foto. Eu fui na direção dela, também bem alterada, falei* (...)*: ‘Você não pode tirar minha foto, então você APAGA minha foto AGORA!’. Então, no que eu fui pra cima dela, eu acho que ela pensou que eu ia tomar o celular dela, ela jogou o celular pra trás e me mordeu*” (Entrevista 9, Técnica em Enfermagem)
Falta de apoio	Banalização do sofrimento do profissional que presta o cuidado de uma situação difícil, por parte da equipe/chefia	“*Na época, tinha um mês que meu pai tinha falecido, então eu já estava EXTREMAMENTE sensível.* (...)*, eu falei com a chefia, mas elas falaram que eu estava sensível demais por causa do meu pai. Mas não foi só isso! mesmo que não fosse meu pai,* (...) *porque foi uma morte HORRÍVEL, entendeu?* (...) *E elas* [a chefia] *não se importaram: ‘Ah, você está assim por causa do seu pai’. E não era só isso! Isso TAMBÉM, isso influenciou, mas não foi só isso’*” (Entrevista 7, Enfermeira)
Trabalho cotidiano com a morte	Trabalho exclusivo com cuidados ao fim de vida, tendo que presenciar episódios de morte quase diariamente	“*A situação difícil, pra mim, foi um escalonamento: ‘não vai parar?’* (...) *em 24 horas eu vi três pessoas morrerem de forma sofrida.* (...) *A situação difícil foi a complexidade de um plantão no qual três pacientes morreram em agonia. A simultaneidade e constância dos acontecimentos, intensificou a situação difícil*” (Entrevista 23, Enfermeira) “*Um paciente foi a óbito e eu preparei o corpo e quando olho para o lado, o outro paciente tinha falecido. De novo, todo aquele trâmite* (...)*. Quando eu vi aqueles dois corpos, naquele saco preto, eu parei no meio daquele quarto, sozinha, fiquei olhando e me peguei falando: ‘Meu Deus do céu! Será que é só isso que vou fazer da minha vida… ficar preparando corpo?*” (Entrevista 10, Técnica em Enfermagem)
Tentativa de suicídio do paciente	Presenciar o paciente atentar contra a própria vida	“*Quando tentou tirar a própria vida, foi uma cena que me marcou bastante.* (...) *ele estava há algum tempo internado, já tinha ocorrido alguns óbitos ao lado dele e no terceiro óbito* (...)*, foi o momento que deu uma surtada* (...) *ele pegou o acesso do soro e colocou em volta do pescoço e fez movimentos de tentar se enforcar...*” (Entrevista 17, Assistente social)

Fonte: elaboração própria.

**Quadro 2:** Tipos, definição e relatos de sentimentos.

CATEGORIA	DEFINIÇÃO	RELATOS DOS ENTREVISTADOS
Impotência	Engloba frustração e sentimento de fracasso	“*É a impotência de não poder curar a pessoa, é tipo: ‘Eu vou aliviar o seu sofrimento, mas apenas o seu sofrimento FÍSICO’*” (Entrevista 1, Enfermeira) “*Impotência diante de uma organização de serviço público, um estado que a gente não consegue atender e abarcar as demandas básicas* (...) *e a frustração, por colocar expectativa* [do casamento] (...) *e naquele momento não foi atendida*” (Entrevista 17, Assistente social)
Angústia	Inclui também o sentimento de ansiedade	“*Isso ia me gerando angústia, porque assim: ‘Caraca! não dá para fazer nada’, porque eu sabia que aquilo* [obstrução] *iria fechar em algum momento e que ela iria morrer asfixiada*” (Entrevista 21, Fisioterapeuta) “*Era angustiante vê-lo daquele jeito, era como se ele tivesse se afogando, sem tá na água...*” (Entrevista 23, Enfermeira)
Compaixão	Engloba empatia, carinho e pena	“*Por que ele está passando por isso?’ E aí, você vê um sentimento de COMPAIXÃO mesmo, entrar no sofrimento dele e tentar de alguma forma amenizar aquilo*” (Entrevista 1, Enfermeira) “*Troquei o curativo inúmeras vezes* (...) *e apesar de ser sempre muito difícil, eu ia fazer com muito carinho, era recompensador dar alívio a ele e mesmo que não soubesse, já havíamos criado um vínculo*” (Entrevista 24, Enfermeira)
Vergonha	Constrangimento e “ser julgado” foram agrupados nessa subcategoria	“*Fiquei muito preocupada com o que as pessoas iam pensar ao meu respeito, porque eu não era aquilo que aconteceu aquele dia* (...) *não me conheci, eu senti vergonha, acho que o maior sentimento foi a vergonha*” (Entrevista 9, Técnica em Enfermagem)
Gratidão	Inclui conforto	“*...porque no momento de despedida, no momento de corte* [morte]*, você ainda assim ter um acompanhante que vem e te agradece.* (...) *então, eu passei pela tristeza, fui à questão da empatia e ao final, eu fiquei feliz pela gratidão*” (Entrevista 2, Enfermeira) “*...por mais que tenha sido sofrido, eu tô com minha consciência tranquila de que eu fiz o que tinha que ser feito. É uma recompensa* (...) *estou ajudando as pessoas, tem algum propósito pra eu estar aqui*” (Entrevista 23, Enfermeira)
Decepção	Inclui a ingratidão	“*Eu fiquei muito decepcionada. Eu falei: ‘eu não fiz nada’, e a pessoa olhar pra mim, e dizer que eu não tô valendo nada?!, Como assim? Eu tô cuidando com carinho*” (Entrevista 3, Técncia em Enfermagem)
Solidão	Reflete o sentimento de desamparo vivido pelos profissionais durante o cuidado	“*A gente se vê sozinho porque a equipe está fragmentada, porque eu não tinha uma* [profissional] *ali no meu lado, que talvez me ajudasse*” (Entrevista 6, Médica) “*Eu me senti MUITO mal de estar naquele quarto* (...)*, por estar ali* (...) *sozinha preparando aqueles corpos*” (Entrevista 10, Técnica em Enfermagem)
Tristeza	O sentimento de mágoa está agrupado nesta subcategoria	“*E eram pacientes que estavam sozinhos e eu acho tão triste quando a pessoa morre sem ter ninguém ao lado, isso me incomoda*” (Entrevista 10, Técnica em Enfermagem) “*...fiquei triste por ela* [mãe]*, de ver uma pessoa perdendo alguém que ama daquele jeito, a forma como ele estava indo embora*” (Entrevista 23, Enfermeira) “*...e o segundo* [sentimento foi] *de tristeza, porque a gente quer fazer mais, a gente quer superar, a gente quer que o outro sorria!*” (Entrevista 12, Médica)
Medo	Agrega sentimento de vulnerabilidade, em relação a si ou a um familiar seu	“*...a gente fica muito vulnerável* (...) *porque era um militar* [acompanhante]*. O pior de tudo, era um militar’.* (...) *poxa, eu tô fazendo tudo!* (...) *você se sente agredida por uma pessoa que está tentando ajudar*” (Entrevista 4, Técnica em Enfermagem) “*Eu acho que senti medo. Medo de perder meu filho. Eu não estou preparada para isso! Não estou preparada para perder meu filho, de jeito nenhum!*” (Entrevista 16, Enfermeira)
Desgaste emocional	Também agrega o sentimento de sofrimento e estar chateada, confusa	“*...pelo nosso desgaste emocional e psíquico* (...)*, ficar aqui, todo dia, trabalhando e lidando com a morte, lidando com a sua finitude, isso te consome, né?*” (Entrevista 15, Enfermeira) “*...porque ao mesmo tempo que eu fiquei chateada por estar cuidando do pai dela de forma respeitosa e não entender por que ela me abordou assim e ao mesmo tempo eu tinha que entender ela. Então eu fiquei confusa, querendo entender o MEU lado e querendo entender o lado dela*” (Entrevista 9, Técnica em Enfermagem)
Raiva	Agrupado com o sentimento de revolta	“*Olha, existe em mim a raiva, e, principalmente, a desvalorização.* (...) *e isso é muito ruim para o profissional, a gente precisa ser reconhecido, nem que seja um ‘obrigado’*” (Entrevista 2, Enfermagem)
Culpa, nojo, alívio	Não foram agrupados, pois não havia conotação semelhante e foram citados de maneira pontual	“*E aí me sentindo culpada porque eu não fiz ela morrer bem*” (Entrevista 15, Enfermeira) “*Senti nojo logo de início,* (...) *fazia o curativo dele com todo o cuidado para que ele não sentisse dor, mesmo estando sedado* (...) *no final, isso me doeu, me deixou triste por sentir nojo... Era um ser humano!*” (Entrevista 24, Enfermeira) “*A gente já esperava que o dia que essa paciente falecesse, ele ia agredir a equipe, mas, GRAÇAS A DEUS! ele não estava no dia, foi um outro familiar que veio. Então, a gente se sentiu um pouco mais aliviado, não por ela ter ido a óbito, mas pela situação, porque a gente esperava o pior*” (Entrevista 4, Técnica em Enfermagem)

Fonte: elaboração própria.

Inicialmente não houve intenção de integrar a categoria profissional na análise, entretanto, por identificarmos diferenças no relato entre as categorias, ela foi incluída na análise, agrupando-as da seguinte forma:

Grupo 1 - Profissionais de saúde que mantém contato direto e contínuo com pacientes. Identificamos que a equipe de enfermagem (técnicos de enfermagem e enfermeiros) apresenta essa característica mais acentuada do que os outros profissionais;

Grupo 2 - Profissionais de saúde que têm contato menos constante com os pacientes. É um cuidado menos intenso e mais pontual, geralmente por demanda. Fazem parte desse grupo os psicólogos, assistentes sociais, nutricionistas e fisioterapeutas;

Grupo 3 - Profissionais que atuam no cuidado, porém nem tão contínuo como o grupo 1 e nem tão pontual como o grupo 2, mas com atribuições indelegáveis. Esse grupo engloba os médicos.

Para manter o anonimato dos entrevistados, substituiu-se os nomes de profissionais ou pacientes por “ciclano” e “fulano”.

Esta pesquisa foi aprovada pelos Comitês de Ética em Pesquisa da Escola Nacional de Saúde Pública Sergio Arouca, Fundação Oswaldo Cruz (protocolo nº 10983019.2.0000.5240) e do Instituto Nacional de Câncer José de Alencar Gomes da Silva (protocolo nº 10983019.2.3001.5274).

## Resultados

### Situações difíceis

As situações difíceis mais frequentes vivenciadas por profissionais de saúde foram: identificação (14); binômio mãe-filho (8); morte ruim (7); divergência de conduta (6); paciente jovem (5) e desfiguração de face (5). Em seguida, vieram: violência contra a equipe (3); tumor de crescimento rápido (3); trabalho cotidiano com a morte (4). Tiveram uma menção como situação difícil no cuidado: medicamento sem efeito (1); perspectiva curativa trabalhando em cuidados paliativos (1); paciente bonzinho (1); falta de apoio (1); tentativa de suicídio de paciente (1).

O processo de identificação, além de ser o principal fator responsável para provocar uma situação difícil, também se mostrou recorrente quando envolvia binômio mãe-filho e paciente jovem. Apesar de ser narrado de forma corriqueira, percebe-se não haver consciência por parte dos profissionais sobre o quanto o processo de identificação incide sobre o cuidado, gerando tantos sofrimentos.

Nos casos de morte ruim, paciente jovem, retardo do efeito do medicamento para diminuir o desconforto respiratório/dor (medicamento sem efeito) ou paciente não aceitar as poucas opções que o profissional tem para tentar reverter o quadro (divergência de conduta) tornavam a situação ainda mais difícil.

A ocorrência da morte ruim representa a antítese do que é preconizado no cuidado paliativo, que é prover uma “boa morte” (morte com alívio da dor e de outros sintomas angustiantes); portanto, não atingir esse preceito é muito sofrido para o trabalhador da saúde. Ainda nesse tocante, certos profissionais que trabalham com cuidado paliativo priorizam tanto a “boa morte”, obtida por uso de sedativo e morfina, que não percebem a possibilidade de significado distinto, podendo até ser contrário, entre o profissional e o paciente, aumentando o sofrimento mútuo. Essas questões também estão envolvidas nas situações difíceis de divergência de conduta.

Outras subcategorias da situação difícil também mostraram relação com o morrer, são elas: trabalho cotidiano com a morte; perspectiva curativa trabalhando em cuidados paliativos; desfiguração de face; falta de apoio; tentativa de suicídio do paciente. Todas elas têm como pano de fundo a morte como problema central: por um cotidiano marcado por ela, seja simultâneo ou não; pela formação profissional que privilegia o discurso biomédico e não engloba a morte enquanto processo de cuidado e nem o suicídio; pela banalização da morte pela chefia; ou mesmo pelo sofrimento de ter que cuidar de um ser humano vivo em estado de decomposição física compatível com um morto.

Dessa forma, duas subcategorias se sobressaem enquanto motivadores para que ocorra uma situação difícil na perspectiva do profissional de saúde: o processo de identificação e aquelas que envolvem certos tipos de morte.

Ainda sobre a perspectiva curativa trabalhando em cuidados paliativos, alguns aspectos acentuam essas situações difíceis, tais como o processo de formação profissional hegemônico embasado na cura e o trabalho em uma unidade hospitalar exclusiva de cuidados paliativos. Essa situação leva a uma práxis marcada pela ausência de casos de “sucesso” no tratamento e pela frequente sensação de que “não fez nada” pelo paciente, perpetuando essa busca curativa.

As subcategorias paciente bonzinho, paciente jovem e tumor de crescimento rápido também apresentam correlação entre si, relacionada com os valores da sociedade, e carregam uma comoção extra, provocando reflexões sobre os sentidos do adoecimento, inclusive morais e religiosas. O paciente bonzinho é aquele não merecedor de sofrimento; o paciente jovem é aquele que não teve “tempo” para merecer o castigo da morte e para colocar em prática os seus planos. O tumor de crescimento rápido representa, no imaginário dos profissionais de saúde, aquele que não dá chances de se buscar um tratamento eficaz contra o câncer que evite a morte, tornando o paciente vítima da própria doença.

Sobre a subcategoria violência contra a equipe, todas envolviam acompanhante e foram narradas pelo grupo 1, que representa profissionais de enfermagem, mostrando o quanto o cuidado paliativo é complexo, pois envolve, na maioria das vezes, uma tríade: profissional-paciente-acompanhante.

Ainda em relação ao grupo 1, o fato de ser a categoria que executa intervenções, nem sempre agradáveis, com contato contínuo e mais próximo com o paciente e acompanhante, potencializa a chance de vivenciar situações difíceis e agressões durante o cuidado. Além disso, em decorrência da dinâmica do trabalho da equipe de enfermagem, que fica responsável por todos os pacientes internados no setor, o quantitativo elevado de pacientes também influencia as chances de vivenciar situações difíceis.

O grupo 2, que engloba profissionais que prestam um cuidado pontual, narrou casos com menor potencial de dificuldade extrema no âmbito do trabalho.

O grupo 3, formado por médicos, teve como principal situação difícil a divergência de conduta. Isso mostra que o médico - categoria com procedimentos exclusivos, que perpassa diferentes fases da vida e atua como dispositivo de normatização de condutas para as outras categorias profissionais -, apesar do espaço de poder na área da saúde, não está protegido do sofrimento pela responsabilidade que exerce, sobretudo no cuidado paliativo.

De forma geral, não observamos diferença entre vivenciar uma situação difícil em relação ao tempo de experiência profissional, ao passo que aspectos institucionais e relacionais potencializaram as situações difíceis. A esse respeito, as questões mais mencionadas pelos entrevistados foram: déficit de profissionais; falta de reunião para discussão dos casos; dificuldade de trabalhar como equipe, além de entraves na comunicação como favorecedores para ocorrência de situação difícil.

### Sentimentos

Os sentimentos mais relatados pelos profissionais de saúde em decorrência das experiências de situação difícil foram: tristeza (18); impotência (15); angústia (10); compaixão (10); e medo (7). Também foram citados os sentimentos de: desespero (4); desgaste emocional (3); vergonha (5); raiva (3); culpa (3); e solidão (3). Outros sentimentos que apareceram foram: nojo (1); gratidão (1); decepção (1); e alívio (1).

Apesar da disparidade, quase a totalidade dos sentimentos decorrentes de uma situação difícil teve analogia a um estado de sofrimento. Gratidão não teve essa conotação e fazia referência a um sentimento de reconhecimento (do acompanhante ou de si pelo cuidado prestado), mas, mesmo assim, vinha permeado por sofrimento pelo momento vivido (situação difícil).

Alívio, nesta pesquisa, não tem uma conotação de ter vislumbrado algo positivo por meio da situação difícil; ao contrário, foi um sentimento relacionado a evitação do acompanhante, com o qual tinha ocorrido a situação difícil.

Os sentimentos de vergonha, culpa e decepção tiveram relação com reprovação, vinda de si mesmo ou do outro.

Tristeza, impotência e angústia foram narradas de forma simultânea na maioria das entrevistas. O sentimento de compaixão, impotência e tristeza também foram mencionados conjuntamente. Em todas as entrevistas em que a solidão foi mencionada, também houve o sentimento de impotência. Desespero apareceu relacionada com a impotência, uma vez que o profissional não conseguia vislumbrar uma solução para aquilo que era difícil para ele. Os resultados mostram que uma situação difícil evoca um misto de sentimentos que denotam bastante sofrimento.

No tocante às relações entre as categorias situação difícil e sentimentos podemos perceber que o processo de identificação provocou o sentimento de medo, pois, à medida que o profissional de saúde presenciava aquela situação, vivenciava o medo de também passar por ela, seja pela perda de um filho jovem ou do(a) companheiro(a).

Morte ruim e medicamento sem efeito, experienciadas por não ter sido capaz de auxiliar o paciente, geraram os sentimentos de impotência, culpa e vergonha, enquanto binômio mãe-filho evocou tristeza entre os profissionais de saúde.

Também pode-se perceber que o sofrimento vivenciado por alguns profissionais estava relacionado com as incertezas do cuidado ao fim da vida, se poderia ter feito algo diferente (conduta não protocolizada) ou se o que fez foi o suficiente para o outro (paciente/acompanhante).

As situações difíceis divergência de conduta, perspectiva curativa trabalhando em cuidados paliativos e tumor de crescimento rápido produziam o sentimento de impotência, uma vez que o profissional se sentia rejeitado em relação à sua proposição terapêutica ou como se não tivesse mais nada a fazer pelo paciente. Também percebemos que violência contra a equipe causava vergonha e medo, seja pelo julgamento do outro, seja pelo receio da agressão física.

## Discussão

De acordo com as situações narradas, podemos perceber que uma experiência difícil para um profissional pode não ser difícil para outros; entretanto, certos acontecimentos do cuidado são difíceis para todos os profissionais. Sobre essa questão, Frota ^10^ aponta que cada profissional de saúde pode ter seus “bichos” ou desafios e, nessa perspectiva, Larrosa ^9^ complementa que a experiência é singular, não repetível, incerta e irredutível. Mas, para que isso ocorra, é necessária uma predisposição para parar, olhar, escutar, pensar devagar e se deter nos detalhes; caso contrário, será uma vivência instantânea, pontual e fragmentada ^9^.

Em relação ao sofrimento vivenciado por profissionais paliativistas, outros autores ^11^^,^^12^^,^^13^^,^^14^ identificaram sentimentos semelhantes, mas significantemente maiores em unidades com ambientes éticos piores e entre a categoria de enfermagem ou aqueles envolvidos diretamente no cuidado, quando comparado com quem presta cuidado mais pontual ^12^.

Os resultados foram similares aos encontrados por Bastos et al. ^13^, à medida que o acompanhante também esteve implicado em situações difíceis, ora nos episódios de violência, ora gerando maior dificuldade para prestar o cuidado de um moribundo perante um familiar, sobretudo em casos de binômio mãe-filho.

O fato de diversas situações difíceis (morte ruim, medicamento sem efeito, trabalho cotidiano com a morte, tentativa suicídio do paciente, desfiguração de face) estarem relacionadas com a morte ou o moribundo pode ser analisada sob a perspectiva de Elias ^6^ e Ariès ^15^^,^^16^.

Para Elias ^6^, ocorreu a transformação da morte de pública (na vida das pessoas) para privada (em hospitais), sendo procedimentos como fechamento de cortinas e lençol para cobrir o corpo, caixão e sepultura, aspectos simbólicos que isolam o moribundo da sociedade e, paulatinamente, estabelecimento da “regra de conduta” de não mostrar o morto e, por consequência, sentimentos. Hospitais exclusivos de cuidado paliativo também podem ser compreendidos nessa perspectiva.

Adicionalmente, o ressurgimento do hospital para morrer (majoritariamente composto por enfermagem) pode ser analisado sob a perspectiva econômico-produtiva ^17^. Para Foucault ^17^, essa transformação evidencia a distinção do valor econômico-social entre indivíduos, por deixar o hospital “médico” exclusivo para indivíduos com perspectiva de cura e, portanto, com potencial produtivo.

Ariès ^15^^,^^16^ salienta a criação empírica de um estilo de morte, como forma de parecer digna e aceitável na sociedade moderna.

Os cuidados paliativos produziram uma normalização médica da morte e introduziram uma nova gestão dela, o modelo da “boa morte”, como consequência, um paradoxo se instala: os aparatos tecnológicos que simbolizam uma medicina desumana, agora são usados para propiciar a “humanização” do morrer ^11^. Com isso, todos os envolvidos - profissionais, pacientes e familiares - devem desempenhar um papel que resulte na produção coletiva da “boa morte” ^11^.

Assim, diversos fatores apartaram a morte e os moribundos da vida na sociedade moderna, pelo medo da própria morte; entretanto, esse afastamento não deixa de provocar dor e sofrimento, visto que, apesar do avanço da medicina, não é possível assegurar a todos uma morte pacífica ou sem aflição ^6^.

O desejo de prover uma “boa morte”, conforme os padrões preestabelecidos pela sociedade e pela formação profissional, cria uma tensão diante da realidade de um evento diverso como a morte. Além de ser mais difícil respeitar o desejo do paciente quando ele recusa um sedativo, por exemplo, uma vez que existe, a priori, um conceito de morrer idêntico para todos, que é sereno.

Para Menezes ^11^, o propósito final dos paliativistas é a “boa morte”, compreendida como aceitação por todos os envolvidos. Nesta pesquisa, o conceito de “boa morte” é antítese da morte ruim e tem relação com acepção simbólica de morte tranquila, e qualquer evento que saia desse modelo (asfixia, contenção mecânica, extravasamento de fluidos, expressão de medo pelo moribundo etc.) é interpretado como sofrimento para o paciente e, por conseguinte, para o profissional. Apesar da diferença sutil, a partir de ambas as interpretações, explica-se o sentimento de impotência quando ela não é alcançada.

Em analogia ao conceito de “boa morte”, estende-se o de paciente bonzinho, que representa aquele que, mesmo doente ou em morte iminente, é condescendente com os padrões morais e culturais de comportamento cooperativo e silencioso, com vistas a manter o ambiente inabalável. Esses padrões impostos ao ambiente hospitalar - harmônico e silencioso - são exigidos, também, dos acompanhantes e profissionais, caso contrário, não seria possível atingi-lo.

A partir do momento em que a morte tumultua o ambiente “tranquilo” do hospital - pelos episódios de dor, sangramento ou fluídos incontroláveis, sufocamento, alucinações, crises de desespero e agonia dos doentes - e o moribundo passa a ser desagradável (“paciente ruim”), a manobra do profissional visa a controlar e eliminar o “inconveniente” na cultura institucional. O objetivo é tornar a morte tolerável para os sobreviventes; nesse caso, familiares e equipe do hospital.

Segundo Ariès ^15^, o moribundo é um ser privado de vontade, de consciência e, por vezes, perturbador. Essa perturbação tem relação com o processo de recalque social acerca das emoções para lidar com a morte ^6^.

Situações de violência contra a equipe podem ter relação com o medo da morte ^6^^,^^16^, vivenciado por familiares/acompanhantes que reagem com agressividade contra quem não obteve êxito na manutenção da vida de um ente querido. Evoca a morte do outro ^16^ que consiste na transferência do medo da própria morte pelo medo de perder alguém amado.

A “boa morte” e a “morte ruim” são processos de construção baseados no impacto sentimental dos hospitais e nos padrões de humor intangíveis que influenciam os sentimentos da enfermagem ^18^. Esses conceitos focaram mais no evento de morte e nas habilidades dos profissionais para gerenciar as demandas organizacionais, do que nas necessidades dos pacientes e no processo de morrer ^18^. Isso mostra o quanto o cuidado está orientado primordialmente para a organização profissional-institucional ^11^ em detrimento daquele a quem o cuidado se destina.

A forma como se quer morrer é uma questão bastante sensível e que necessita ser aprofundada, especialmente porque o princípio da autonomia repousa em diversos pressupostos que limitam sua aplicabilidade à beira do leito ^19^, e não são raras as vezes em que as decisões quando os pacientes estão relativamente saudáveis divergem de quando a morte é iminente ^20^.

Para Siebers ^5^, a dor é representada no imaginário social como um sofrimento temido e indigno, sendo assim, a “morte ruim” é aquela que não confere dignidade humana para a sociedade. O autor ^5^ salienta que a dor é subjetiva, de difícil compreensão e não é igual para todos, mas mesmo que não se saiba exatamente o que o outro sente, é comum imputar sentimentos de dor e sofrimento a terceiros, levando em conta somente a aparência ou as circunstâncias. E complementa afirmando que a dor pode provocar fortes emoções, opiniões e julgamentos pela falta de reflexão contextualizada.

Esse entendimento é fundamental para compreender a situação difícil divergência de conduta. Questões subjetivas, como a dor e o sofrimento, que mobilizam fortes emoções tendem a ter como consequências ações voltadas para os próprios conceitos e ideais, sem levar em consideração o outro.

Segundo Menezes ^11^, tanto médicos curativistas quanto paliativistas atuam de forma onipotente e sem consciência dos limites do exercício profissional, assim, apregoa que não há ruptura da racionalidade médica pelos paliativistas. A diferença é que os últimos buscam o modelo “contemporâneo” de morrer que, para autora, é uma sofisticação do poder médico mediado pelo trabalho em equipe.

Na lógica de cuidado tecnocrática, em contraposição ao intersubjetivo, não há espaço para o saber do outro. Assim, o saber técnico se sobrepõe às experiências, aos valores, às crenças e aos conhecimentos de quem recebe o cuidado ^21^, gerando maiores possibilidades de haver conflito por divergência de conduta.

A situação difícil perspectiva curativa trabalhando em cuidados paliativos também apresenta a mesma dinâmica e, além disso, pode ser explicada pela atuação do modelo biomédico hegemônico sobre o processo de formação profissional, de organização da assistência e da função gerencial ^21^.

No caso de desfiguração da face, tumores extensos e aparentes carregam um imaginário de sofrimento e dor, provocando uma enorme carga emocional, tal como enfatizado por Siebers ^5^. Casos em que ocorre desfiguração da face carregam o simbolismo identitário do corpo humano e da morte estampada no rosto do outro, que não se pode ocultar ^6^, aumentando a impotência de trabalhadores formados sob a égide da cura e do adiamento da vida, isto é, com perspectiva curativa trabalhando em cuidados paliativos.

A dimensão subjetiva do cuidado compreende que, ao se deparar com alguma desordem orgânica, cada indivíduo a experimenta de maneira única e particular. Sendo assim, a visão dos profissionais deve ser voltada para tal questão. Isso não quer dizer que devemos esquecer os aprendizados técnicos, mas esses não podem ser sobrepostos ao que realmente importa e faz sentido ao trabalho em saúde: o cuidado individual do outro.

Tumores de crescimento rápido e pacientes jovens remetem ao sentimento de vulnerabilidade dos indivíduos diante da doença e da morte, propiciando medo pela própria morte e de seus entes queridos, além de gerar compaixão. Em situações antagônicas, ou seja, tumores de crescimento lento, relacionados ao estilo de vida, podem ter um efeito oposto, sendo esses pacientes, em geral, vistos como socialmente inadequados e negligentes e frequentemente responsabilizados pela própria doença. Consequentemente, pacientes considerados não merecedores podem despertar a compaixão, o cuidado que ameniza. Paciente bonzinho e binômio mãe-filho também seguem essa analogia de “não merecedores”. Porém, caso sejam considerados moralmente culpados pela doença, o cuidado pode se tornar distante. Sontag ^8^ chama a atenção para como a imposição das ideias moralizantes judaico-cristãs no cuidado estabeleceu uma ligação metafórica de punição para a doença; assim, alguns pacientes seriam considerados culpados, portanto, promovedores da doença, e outros, vítimas, em relação aos comportamentos adotados por eles.

Chan et al. ^14^ apontaram que profissionais de saúde sentem dificuldade de compreender o motivo de coisas ruins acontecerem com pacientes de bom coração.

Ainda sobre essas considerações das situações difíceis binômio mãe-filho e paciente jovem, também são marcadas por um cuidado impregnado de crenças morais judaico-cristãs, nas quais morrer jovem ou perder a mãe em idade jovem, gera piedade profissional e sentimento de injustiça divina. Por esse motivo, essa morte é mais sofrida: uma vida sem ter sido vivida ^22^ ou viver desde cedo desprovido da proteção materna.

O cuidado intersubjetivo chama a atenção para as possíveis divergências de valores e crenças entre profissional e paciente/acompanhante, reforçando a necessidade de compreender a cultura e saber do outro, favorecendo que os dois “universos” consigam interagir, sem se anular.

Um estudo específico com oncologistas apontou que, quando eram pacientes jovens, de idade semelhante à dos médicos ou que viviam momentos parecidos em suas vidas, havia maior dificuldade de interromper o tratamento quimioterápico, mesmo que não houvesse benefício dele, e encaminhar para o cuidado paliativo ^22^. Esses achados ratificam os resultados encontrados, mostrando evidências de maior sofrimento no cuidado paliativo quando diante de paciente jovem e do processo de identificação.

Laryionava et al. ^22^ também observaram que o cuidado é atravessado por forças inconscientes e pelo processo de identificação. Nesse tocante, os profissionais mais jovens eram mais vulneráveis ao comportamento de identificação com pacientes em situações de vida parecidas do que os oncologistas mais idosos.

A respeito das limitações do estudo, destacamos que foi realizado em hospital exclusivo de cuidados paliativos oncológicos, o que pode ter gerado situações difíceis mais intensas e relacionadas à morte. Contudo, acreditamos que situação difícil e sofrimento são importantes dimensões do cuidado em outros níveis assistenciais e a outras patologias.

A título de reflexão, esperava-se que o fato de as entrevistas serem conduzidas por uma profissional da mesma unidade assistencial pudesse inibir certas “fragilidades” dos profissionais ou evidenciasse experiências de autopromoção. Felizmente, foi justamente o contrário. Possivelmente pela sensibilidade do tema proposto, por ser aquele silenciado dentro dos serviços de saúde, contribuiu para elevar a confiança na entrevistadora.

## Conclusão

Apoiada em experiências ^9^ de situações difíceis de profissionais do cuidado paliativo, a pesquisa partiu do princípio de que a modificação do estado interno do profissional no cotidiano do seu cuidado possibilita a experiência, que gera uma transformação e um novo significado e saber a partir da práxis.

Desse modo, a partir do resultado de 30 situações difíceis compartilhadas, pudemos compreender, de forma resumida, que as principais situações difíceis foram decorrentes de processo “inconsciente” e que permaneceram dolorosas por longo período. Os principais motivos foram o processo de identificação do profissional com o paciente e suas histórias de vida, além de contextos que envolviam binômio mãe-filho, paciente jovem e morte ruim, as quais têm uma relação com conjunto de crenças e valores morais da sociedade em que vivemos. Dentro do contexto do cuidado paliativo, a violência no cuidado causou surpresa, uma vez que eram esperadas apenas situações relacionadas ao processo de fim de vida.

Diversos sentimentos dolorosos, como a tristeza, angústia, medo, solidão, impotência, desespero, nojo, entre outros, emanaram dessas situações, mas também foram relatados sentimentos de gratidão e compaixão. Esses resultados mostram que o cuidado suscita uma pluralidade de sentimentos, a maioria desagradáveis, denotando o grau de sofrimento dos profissionais. Sentimentos agradáveis tinham relação estreita com a sensação de ter feito o seu melhor e ter sido reconhecido por isso.

Evidenciou-se, também, o efeito sobre o processo de morrer ^6^^,^^15^^,^^16^, decorrente do contínuo processo de ocultação, demarcado pelo afastamento entre vivos e moribundos ^6^ e pelas rotinas instituídas pelas organizações de saúde ^6^^,^^16^, tornando angustiante seu processamento para todos os envolvidos, inclusive aqueles que atuam no cuidado paliativo.

Compreendemos que o cuidado pode proporcionar diversas situações aos profissionais; entretanto, quando é ofertado em uma modalidade em que a morte está constantemente presente, essas experiências se intensificam, mobilizando reflexões intimamente relacionadas com sua própria finitude e dos seus entes.

Por fim, os resultados desafiam os profissionais a focar a atenção na morte como um processo e a priorizar as necessidades dos pacientes acima do das instituição ^11^^,^^18^, permitindo que pacientes tenham um papel na definição de eventos no final de suas vidas.
